# Left Atrial Appendage Closure With Catheter Ablation vs. Ablation Alone on Outcomes of Atrial Fibrillation in Heart Failure With Reduced Ejection Fraction: A Propensity Score-Matched Analysis

**DOI:** 10.7759/cureus.74577

**Published:** 2024-11-27

**Authors:** Fidelis E Uwumiro, Oghenemaro O Oghotuoma, Nathaniel Eyiah, Somto Ojukwu, Gentle C Uwaoma, Victory Okpujie, Temabore V Daboner, Justice C Mgbecheta, Claire A Ewelugo, Ifeanyi Agu, Omolade Oshodi, Stanley S Ezulike, Afeez O Ogidan

**Affiliations:** 1 Internal Medicine, Prime Healthcare-SRGA, Riverdale, USA; 2 General Internal Medicine, Walsall Healthcare NHS trust, Walsall, GBR; 3 Internal Medicine, University of Cape Coast School of Medical Sciences, Cape Coast, GHA; 4 Internal Medicine, Ebonyi State University Teaching Hospital, Abakaliki, NGA; 5 Internal Medicine, College of Medicine, University of Nigeria, Enugu, NGA; 6 Internal Medicine, Central Hospital Benin, Benin City, NGA; 7 Internal Medicine, Kwame Nkrumah University of Science and Technology, Kumasi, GHA; 8 Internal Medicine, Nuvance Health Vassar Brothers Medical Center, Poughkeepsie, USA; 9 Internal Medicine, Federal University Teaching Hospital, Owerri, NGA; 10 Internal Medicine, Imo State University College of Medicine, Imo State, NGA; 11 Internal Medicine, Kettering General Hospital, Kettering, GBR; 12 Internal Medicine, Chukwuemeka Odumegwu Ojukwu University Teaching Hospital, Awka, NGA; 13 Internal Medicine, Olabisi Onabanjo University, Ago-Iwoye, NGA

**Keywords:** atrial fibrillation, catheter ablation, electrocardiogram (ekg) atrial fibrillation (a-fib) with rapid ventricular response (rvr), heart failure, left atrial appendage closure

## Abstract

Background

Combining left atrial appendage closure with catheter ablation (LAACCA) has been proposed as a potential approach to improving outcomes by simultaneously addressing arrhythmia and reducing stroke risk. This study compares the in-hospital outcomes of LAACCA vs. catheter ablation (CA) alone for atrial fibrillation (AFib) in patients with heart failure with reduced ejection fraction (HFrEF).

Methods

We analyzed adult hospitalizations with HFrEF and AFib who underwent LAACCA or CA alone from the 2016-2020 nationwide inpatient sample using validated ICD-10 codes. Propensity score matching, accounting for patient-, hospital-, and procedure-level covariates, illness severity, and baseline risk of mortality, was used to alleviate bias in nonrandomized treatment assignments. The primary endpoints included all-cause in-hospital mortality, hospital stay, and hospitalization costs. Secondary endpoints included postprocedural complication rates. Prolonged hospitalization was defined as hospital stay in the top decile of hospital stay in each cohort. All statistical analyses in the study were based on weighted hospital data.

Results

About 233,865 HFrEF patients were hospitalized for AFib. Approximately 27,945 (11.9%) underwent LAACCA, while 205,920 (88.1%) underwent CA only. The cohort comprised mostly males (151,077; 64.6%) (mean age: 67.4; SD: 4.3). The propensity score-matched cohort comprised 18,195 LAACCAs and 18,195 CAs; all covariate imbalances were alleviated. LAACCA was associated with a higher rate of prolonged hospital stay (7.6 vs 5.6 days; P<0.001), a higher mortality rate (209 (1.1%) vs. 160 (0.9%); P=0.011), and higher mean hospital costs ($289,960 vs. $183,932; P<0.001) compared with CA alone. LAACCA was associated with a higher incidence of acute myocardial ischemia (528 (2.9%) vs. 455 (2.5%); P=0.013), complete atrioventricular block (1,200 (6.6%) vs. 892 (4.9%); P=0.004), need for implantable device therapy (1,510 (8.3%) vs. 1,348 (7.4%); P=0.017), pneumothorax (328 (1.8%) vs. 91 (0.5%); P<0.0001), hemothorax (200 (1.1%) vs. 127 (0.7%); P<0.0001), pneumonia (983 (5.4%) vs. 546 (3.0%); P<0.0001), vascular access complications (346 (1.9%) vs. 255 (1.4%); P=0.046), and septicemia (309 (1.7%) vs. 182 (1.0%); P<0.001). CA was associated with a greater incidence of cardiac tamponade (237 (1.3%) vs. 382 (2.1%); P=0.010) and femoral artery pseudoaneurysm (364 (0.2%) vs. 91 (0.5%); P<0.001).

Conclusion

LAACCA was correlated with higher mortality odds compared to CA alone for atrial fibrillation in HFrEF.

## Introduction

Atrial fibrillation (AFib), the most common sustained arrhythmia globally and in the United States [1,2] affects approximately 1-3% of the general population [1,3,4]. It manifests as an irregular and often rapid heart rhythm, which is characterized on an ECG by absent P-waves, irregular R-R intervals, and visible fibrillatory waves [5]. Its incidence is notably increasing in older age groups, males, and non-Hispanic White populations [6].

AFib and heart failure (HF) with reduced ejection fraction (HFrEF) can occur independently, but often coexist in the same patient, complicating each other’s course. Their co-existence leads to increased all-cause mortality, higher ischemic stroke incidence, and more frequent hospitalizations [7]. Research indicates that AFib can lead to HFrEF, and vice versa: the rapid heart rate in AFib shortens diastolic filling time, reducing cardiac output, and accelerating HFrEF progression through mechanisms such as tachycardia-induced cardiomyopathy and worsening of hemodynamics - the "AFib begets heart failure" paradigm [8]. In HFrEF, ventricular dysfunction leads to elevated left atrial pressures and volume overload, causing atrial stretch and dilation. This process, coupled with myocardial fibrosis, promotes structural remodeling and the development of reentrant circuits, which are key drivers of AFib.

Additionally, neurohormonal activation, including increased sympathetic tone and renin-angiotensin-aldosterone system (RAAS) activity, exacerbates atrial remodeling. These factors make patients with HFrEF particularly prone to developing or sustaining AFib. Both conditions also share common risk factors, including hypertension, diabetes, obesity, and ischemic heart diseases [7]. The CASTLE-AFIB trial has demonstrated that catheter ablation (CA) is more effective in achieving rhythm control than pharmacological antiarrhythmics in managing AFib in patients with HFrEF [7,9]. Catheter ablation improves quality of life, reduces the burden of AFib, lowers mortality associated with HFrEF, and decreases stroke risk [10-12]. Despite these benefits, there remains a high recurrence rate of AFib after CA, particularly in patients with symptomatic paroxysmal or persistent AFib.

The left atrial appendage, because of its structure and potential for blood flow stasis, is a common site for thrombus formation. Consequently, it is the source of approximately 90% of emboli in nonvalvular AFib, increasing the risk of thromboembolic ischemic stroke in patients with AFib [13-15]. Left atrial appendage closure (LAAC) can be beneficial in reducing stroke risk and decreasing the need for long-term anticoagulation. The LAAC procedure, often using a device such as the endocardial Watchman® (Boston Scientific, Natick, USA), successfully occludes the left atrial appendage with a 98.5% success rate [16]. CA and LAAC are associated with distinct complication profiles. CA, which targets arrhythmogenic foci in the atria, has a complication rate of 4-10%, with common adverse events including pericardial effusion, cardiac tamponade, vascular access complications, and rare risks of pulmonary vein stenosis or stroke [17].

Conversely, LAAC, designed to prevent thromboembolism in patients with non-valvular AFib, carries a complication rate of 2-5%, including device-related thrombus, periprocedural stroke, cardiac perforation, and pericardial effusion [18,19]. Both procedures are generally safe when performed by experienced operators, but patient-specific risk factors, such as age, comorbidities, and procedural complexity, influence outcomes.

In theory, combining LAAC with CA (LAACCA) in a "one-stop" procedure could improve clinical outcomes for AFib, including reducing thromboembolism and AFib recurrence [16]. However, there is insufficient evidence comparing the short-term outcomes of both procedures in patients with HFrEF. Our study aims to fill this knowledge gap by comparing the in-hospital outcomes of LAACCA and CA alone. This study adopted a propensity score-matching approach with a nationwide database to compare LAACCA with CA alone in terms of all-cause in-hospital mortality, duration of hospital stay, and hospital costs. Secondary endpoints included the incidence of in-hospital complications.

## Materials and methods

Study data and selection criteria

This study used data from the nationwide inpatient sample (NIS) database. The NIS, under the egis of the Agency for Health Care Research and Quality (AHRQ), is part of the Healthcare Cost and Utilization Project (HCUP). It is the largest public inpatient database in the United States. NIS data represents approximately 8 million unweighted hospital stays annually across 4,000 hospitals since 2012 and, when weighted, mirrors approximately 35 million annual hospitalizations nationwide each year. The NIS uses a stratified sampling method to select hospitals based on location, teaching status, and size, providing detailed information on healthcare utilization, including length of stay (LOS), total hospital charges (THC), and patient outcomes. The database comprehensively records up to 40 diagnoses and 15 procedures per hospital stay, with each case anonymized and recorded as a unique observation. It includes detailed patient demographics, comorbidities, procedures, insurance details, and in-hospital outcomes and allows for multiyear analysis [20-22]. Starting in 2016, the NIS has recorded a full year’s hospitalization data exclusively using the International Classification of Diseases, Tenth Revision, Clinical Modification and Procedure Coding System (ICD-10-CM/PCS). This system has been consistent since its adoption on October 1, 2015, ensuring uniformity in diagnostic and procedural coding for our analysis.

**Figure 1:**
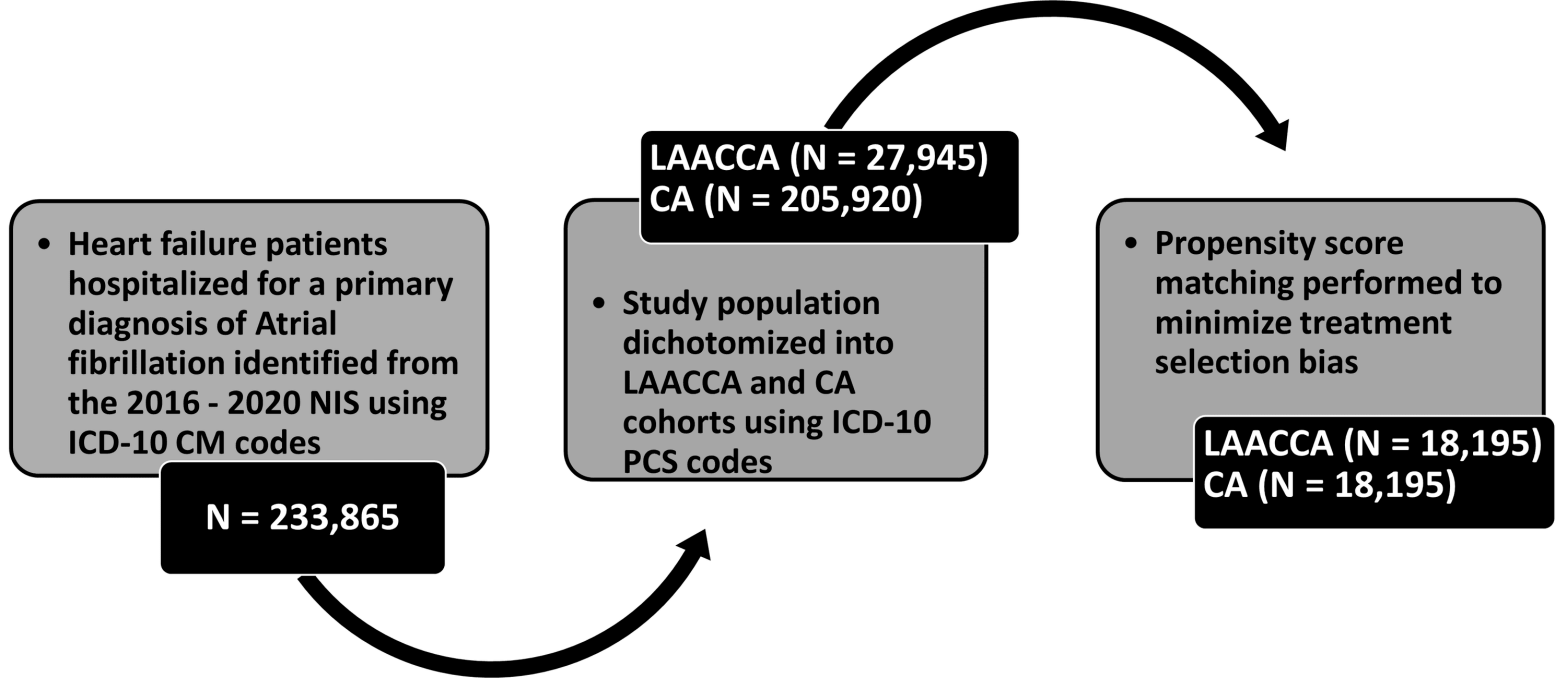
Study cohort selection NIS = nationwide inpatient sample database; ICD-10 CM = international classification of diseases, tenth revision, clinical modification; LAACCA = combined left atrial appendage closure and catheter ablation; CA = catheter ablation only; ICD-10 PCS = international classification of diseases, tenth revision, procedure coding system

The 2016-2020 NIS database was queried for all adult hospitalizations with a primary diagnosis of AFib and a secondary diagnosis of HFrEF using ICD-10 diagnostic codes (I50.20, I50.21, I50.23, and I50.22). Hospitalizations associated with LAAC (ICD-10 procedure codes: 02L70CK, 02L70DK, 02L70ZK, 02L73CK, 02L73DK, 02L73ZK, 02L74CK, 02L74DK, 02L74ZK) or CA (ICD-10 procedure codes: 02580ZZ, 02583ZZ, 02584ZZ) were subsequently identified using the ICD-10 procedure codes. The total cohort was subsequently divided into two groups: hospitalizations with ICD-10 procedure codes for both LAAC and CA (LAACCA) in the index hospitalization and those with CA alone for comparison. Our coding approach is in line with the methodologies established in prior research [23]. For code validation, two authors independently reviewed the ICD-10-CM codes, with a discrepancy rate of 2%. Discrepancies in coding were resolved by consulting a third author, leading to further code refinement and comparison with existing studies. To enhance study reliability, we used the study design checklist for studies based on the NIS proposed by Khera R. et al. in 2017 [24]. The algorithm for study selection is summarized in Figure 1.

Institutional review board approval was waived for this study because the NIS is a public, deidentified database with prior ethical committee approval. The index study relies on anonymous data, thus precluding the need for individual consent. All pertinent data supporting this study have been included in the manuscript and supplementary materials.

Outcomes

The primary outcomes of interest were all-cause in-hospital mortality, THC, and LOS. Mortality is recorded as a dichotomous variable within the NIS, whereas length of stay and total hospital costs (in US$) are recorded as continuous variables. Secondary outcomes included in-hospital complications such as cardiac perforation or cardiac tamponade, acute myocardial infarction, complete atrioventricular (AV) block, sick sinus syndrome, need for implantable device therapy (including pacemakers insertion, implantable cardioverter-defibrillators, and cardiac resynchronization therapy), pneumothorax, hemothorax, pneumonia, acute ischemic stroke, phrenic nerve palsy, vascular complications such as hematoma, seroma, and hemorrhage, thromboembolism, femoral artery pseudoaneurysm, oxygen supplementation, and sepsis, which were all identified using ICD-10 codes from the secondary diagnoses variables within the NIS (supplementary data).

Statistical analysis

This study was designed to compare the short-term in-hospital outcomes of LAACCA with those of CA in terms of mortality, LOS, THC, and complications. Continuous variables are expressed as mean (SD), whereas categorical variables are presented as absolute numbers and percentages. For univariate comparisons, Pearson’s χ2 analysis or Fisher exact tests were used to evaluate categorical variables; alternatively, continuous variables were analyzed using t-tests and Wilcoxon rank-sum test for normally and nonnormally distributed data, respectively. We performed a two-step analysis using unmatched and matched LAACCA and CA hospitalizations. In the unmatched analysis (primary analysis), stepwise, univariable logistic regression analyses identified covariates associated with primary and secondary outcomes of interest (P ≤ .05 for entry; P > 0.10 for removal). Subsequent multivariable logistic regression analysis was conducted accounting only for factors significantly associated with outcomes of interest, including the following covariates: age, sex, Charlson comorbidity index, illness severity, race, insurance status, hospital region, type of admission, and comorbidities. Multicollinearity was assessed by computing the matrix of correlation between covariates using the variance inflation factor (VIF). Covariates with VIF values >5 were excluded from the regression analysis, maintaining a simpler model without compromising model accuracy.

Propensity score matching is a method used to minimize treatment selection bias when estimating causal treatment effects in nonrandomized studies [25]. In the index study, “Control” (i.e., CA) and “case” (i.e., LAACCA) cohorts were matched on a set of variables that would otherwise confound comparisons between them. Once a matched sample was formed, the treatment effect was estimated by directly comparing the primary and secondary outcomes between controls and cases in the matched sample [26]. In the secondary analysis (matched model), propensity scores were developed accounting for all factors significantly different between the LAACCA and CA cohorts in the unmatched model or significantly associated with undergoing LAACCA on logistic regression analysis. Accordingly, individual propensity scores were calculated through logistic regression modeling [27] based on the following covariates: age, sex, insurance, race, type of admission (elective vs. nonelective), Charlson comorbidity index (CCI), previous myocardial infarction, percutaneous coronary intervention (PCI) or coronary artery bypass grafting (CABG), weekend vs. weekday admissions, illness severity, hospital region, and comorbidities (obesity, dyslipidemia, coronary artery disease, obstructive sleep apnea, peripheral vascular disease, cerebrovascular disease, chronic obstructive pulmonary disease (COPD), dementia, liver, renal or rheumatoid disease and patient location). The LAACCA and CA patients were then paired 1:1 on these propensity scores using exact matching [28]. A standard caliper size of 0.2 × log (SD of the propensity score) was used. Standardized differences were estimated before and after matching to evaluate the balance of covariates; small absolute values (<0.1) indicate the balance between the treatment groups.

The post-match balance of the covariates was assessed graphically and statistically. Following 1:1 propensity score matching, the occurrence of mortality and complications between matched LAACCA and CA patients was examined using the McNemar test. Continuous outcomes, such as length of hospital stay and total hospital costs, were compared between the matched LAACCA and CA cohorts using paired sample t-testing or Wilcoxon rank-sum test. P ≤ .05 was considered statistically significant; all tests were two-sided. All statistical computations were based on weighted nationwide inpatient data and were performed using Stata statistical software (version7.0MP; Stata Corp LLC, College Station, USA) with propensity score matching performed using the Stata extension program developed by Edwin Leuven and Barbara Sianesi [29].

## Results

A total of 233,865 patients with HF who were hospitalized for a primary diagnosis of AFib and who underwent LAACCA or CA were analyzed. Approximately 27,945 hospitalizations underwent LAACCA, whereas 205,920 underwent CA only. The overall cohort comprised 151,077 (64.6%) males and had a mean age of 67.4 (SD, 4.3) years.

Factors predicting undergoing LAACCA

Before propensity score matching, significantly different variables on univariable comparisons between the LAACCA and CA cohorts were entered into a multivariable logistic regression model to identify predictors of undergoing LAACCA. Variables significantly correlated with undergoing LAACCA included: older age (adjusted odds ratio (aOR): 1.10; 95% CI, 1.08-1.14; P < 0.001), more co-morbidities with CCI score = 1 (aOR: 5.86; 95% CI, 3.24-10.61; P< 0.001), CCI score = 2 (aOR: 10.6; 95% CI, 5.87-16.1; P < 0.001), CCI score = 3 (aOR: 19.2; 95% CI: 10.80-14.26; P < 0.001), and presence of old myocardial infarction (old MI) (aOR, 1.45; 95%CI, 1.19-1.76; P < 0.001). Previous CABG was associated with lower odds of LAACCA (aOR: 0.68; 95% CI: 0.50-0.92; P = 0.001).

Characteristics of the propensity score-matched cohorts

To more effectively control for confounding factors when selecting patients for combined left atrial appendage closure and catheter ablation vs. catheter ablation alone, we matched patients in a 1:1 ratio. This matching was based on the predictors of increased likelihood of undergoing LAACCA, covariates significantly different between both groups in the unmatched cohorts (P-value<0.005; Table 1), and factors associated with the occurrence of clinically relevant postprocedural outcomes in the unmatched cohort. The propensity score-matched cohort from our primary analysis included 36,390 patients, with an equal distribution of 18,195 patients (50.0%) in both the LAACCA and CA groups. The previously noted differences in patient- and hospital-level covariates between the LAACCA and CA cohorts were significantly reduced after matching. These differences included factors such as age, sex, Charlson comorbidity index (CCI) scores, race, primary payer, hospital region, weekend vs. weekday hospitalizations, elective vs. nonelective admissions, patient location, disease severity, and comorbidity burden. The matching process proved effective, reducing the absolute standardized differences for all covariates to less than 10%. Table 1 summarizes demographic characteristics before and after propensity score matching.

**Table 1:** Comparison of patient- and hospital-level covariates between cohorts undergoing LAACCA or CA before and after propensity score matching The data is presented as frequency (N) and percentages (%) for all categorical variables whereas age data is summarized median with interquartile range (IQR) Values are N (%) unless indicated otherwise HF, heart failure; CCI, Charlson comorbidity index; MI, myocardial infarction; COPD, chronic obstructive pulmonary disease; CCF, congestive cardiac failure; CABG, coronary artery bypass grafting; PCI, percutaneous coronary intervention; HMO, health maintenance organization; LAACCA, combined left atrial appendage closure and catheter ablation; CA, catheter ablation alone; LOF, loss of function; * Significant at values <0.001 ** Sundararajan's adaptation of the modified Deyo's Charlson Comorbidity Index (CCI), which offers a refined approach for population-based investigations. This adaptation classifies the CCI into four distinct groups, each indicative of escalating mortality risk. A CCI score of ≥3 is associated with high comorbidity burden and an approximate 25% 10-year mortality rate, whereas scores of 2 or 1 correspond to mild to moderate comorbidity burden with 10% and 4% 10-year mortality rates respectively. A score of 0 implies no significant comorbidity.

Variable	N (%) unless otherwise specified					
	Before Propensity Score Matching			After Propensity Score Matching		
	LAACCA (N = 27,945)	CA (N = 205,920)	P -value*	LAACCA (N = 18,195)	CA (N = 18,195)	P -value*
Median age, years (IQR)	69 (62-75)	68 (59-76)	<0.001	68.2 (61-72)	68.3 (59-71)	0.657
Sex			<0.001	Sex		0.950
Male	18,863 (67.5)	127,053 (61.7)	-	11,827 (65.0)	11,827 (65.0)	-
Female	9,082 (32.5)	78,867 (38.3)		6,368 (35.0)	6,368 (35.0)	
Insurance type			<0.001	Insurance type		0.538
Medicare	18,443 (66.0)	130,553 (63.4)	-	11,881 (65.3)	11,936 (65.6)	-
Medicaid	1,341 (4.8)	15,238 (7.4)		928 (5.1)	1,019 (5.6)	
Private, including HMO	7,825 (28.0)	56,216 (27.3)		5,167 (28.4)	5,313 (29.2)	
Self-pay	335 (1.2)	3,912 (1.9)		218 (1.2)	309 (1.7)	
Race			<0.001	Race		0.822
White	24,060 (86.1)	160,412 (77.9)	-	15,502 (85.2)	14,884 (81.8)	-
Black	1,174 (4.2)	22,033 (10.7)		892 (4.9)	1,510 (8.3)	
Hispanic	1,285 (4.6)	13,179 (6.4)		873 (4.8)	1,019 (5.6)	
Asian/Pacific Islanders	671 (2.4)	3,707 (1.8)		437 (2.4)	364 (2.0)	
Native Americans	112 (0.4)	824 (0.4)		73 (0.4)	55 (0.3)	
others	671 (2.4)	5,766 (2.8)		473 (2.6)	382 (2.1)	
Nonelective admissions	19,813 (70.9)	73,926 (35.9)	<0.001	11,718 (64.4)	11,827 (65.0)	0.386
Weekend admissions	1,900 (6.8)	30,270 (14.7)	<0.001	1,564 (8.6)	15,830 (8.7)	0.272
All Patient Refined-DRG (APR-DRG): Severity of illness			<0.001	APR-DRG: Severity of illness		0.010
Minor LOF	12,184 (43.6)	10,708 (5.2)	-	1,874 (10.3)	3,384 (18.6)	-
Moderate LOF	4,946 (17.7)	108,932 (52.9)		3,657 (20.1)	6,714 (36.9)	
Major LOF	7,880 (28.2)	74,748 (36.3)		8,442 (46.4)	5,822 (32.0)	
Extreme LOF	2,934 (10.5)	11,531 (5.6)		4,239 (23.3)	2,365 (13.0)	
CCI**			<0.001	CCI		0.456
0	4,751 (17.0)	42,420 (20.6)	-	3,147 (17.3)	3,748 (20.6)	-
1	7,294 (26.1)	46,126 (22.4)		4,713 (25.9)	4,076 (22.4)	
2	5,813 (20.8)	37,889 (18.4)		3,748 (20.6)	3,366 (18.5)	
≥3	10,088 (36.1)	79,691 (38.7)		6,587 (36.2)	7,005 (38.5)	
Hospital region			<0.001	Hospital region		0.913
Northeast	4,220 (15.1)	44,273 (21.5)	-	3,039 (16.7)	3,039 (16.7)	-
Midwest	7,769 (27.8)	44,067 (21.4)		3,967 (21.8)	4,003 (22.0)	
South	10,703 (38.3)	87,310 (42.4)		8,060 (44.3)	8,024 (44.1)	
West	5,282 (18.9)	30,476 (14.8)		3,130 (17.2)	3,130 (17.2)	
Comorbidities						
Obesity	4,695 (16.8)	28,623 (13.9)	<0.001	2,638 (14.5)	2,638 (14.5)	>0.99
Hypertension	9,501 (34.0)	68,983 (33.5)	0.461	6,186 (34.0)	6,350 (34.9)	>0.99
Dyslipidemia	14,811 (53.0)	95,546 (46.4)	<0.001	8,734 (48.0)	8,734 (48.0)	>0.99
Coronary artery disease	9,250 (33.1)	74,543 (36.2)	0.001	6,114 (33.6)	6,004 (33.3)	0.252
Obstructive sleep apnea	6,651 (23.8)	44,891 (21.8)	0.001	4,294 (23.6)	4,403 (24.2)	0.337
Tobacco use	643 (2.3)	5,972 (2.9)	0.474	-	-	-
Old PCI	2,403 (8.6)	22,033 (10.7)	<0.001	1,674 (9.2)	1,565 (8.6)	0.242
Old CABG	475 (1.7)	19,356 (9.4)	<0.001	400 (2.2)	346 (1.9)	0.529
Old MI	5,114 (18.3)	33,565 (16.3)	0.001	3,366 (18.5)	3,257 (17.9)	0.211
Peripheral vascular disease	4,583 (16.4)	36,654 (17.8)	0.016	2,820 (15.5)	3,075 (16.9)	
CCF	15,174 (54.3)	114,080 (55.4)	0.212	10,025 (55.1)	10,025 (55.1)	>0.99
Previous ischemic stroke	2,319 (8.3)	9,472 (4.6)	<0.001	1,001 (5.5)	1,000 (5.5)	>0.99
COPD	5,896 (21.1)	51,686 (25.1)	<0.001	3,985 (21.9)	3,912 (21.5)	0.132
Dementia	252 (0.9)	4,736 (2.3)	<0.001	182 (1.0)	218 (1.2)	0.610
Types 1 and 2 diabetes	8,495 (30.4)	63,012 (30.6)	0.851	5,531 (30.4)	5,422 (29.8)	
Chronic liver disease	643 (2.3)	5,972 (2.9)	0.008	455 (2.5)	473 (2.6)	
Malignant neoplasia	531 (1.9)	6,589 (3.2)	<0.001	382 (2.1)	400 (2.2)	0.560
Chronic renal disease	6,064 (21.7)	48,185 (23.4)	0.007	3,966 (21.8)	4,276 (23.5)	
Rheumatoid disease	615 (2.2)	5,560 (2.7)	0.027	382 (2.1)	528 (2.9)	
Patient location			<0.001	Patient location		0.311
“Central” counties of metro areas of ≥1million population	6,958 (24.9)	58,275 (28.3)	-	4,840 (26.6)	4,840 (26.6)	-
“Fringe” counties of metro areas of ≥1 million populations	6,511 (23.3)	56,422 (27.4)		4,276 (23.5)	4,694 (25.8)	
Counties in metro areas with 250,000–999,999 people	6,651 (23.8)	42,831 (20.8)		4,203 (23.1)	4,003 (22.0)	
Counties in metro areas with 50,000–249,999 people	2,739 (9.8)	17,915 (8.7)		1,747 (9.6)	1,692 (9.3)	
Micropolitan counties	2,683 (9.6)	16,680 (8.1)		1,729 (9.5)	1,729 (9.5)	
Not metropolitan or micropolitan counties	2,403(8.6)	13,591 (6.6)		1,419 (7.8)	1,274 (7.0)	

Effect of LAACCA vs. CA on primary outcomes in the propensity score-matched cohort

Secondary outcomes were compared in both cohorts prior to propensity score matching (Table 2).

**Table 2:** Logistic regression model for clinical outcomes in 233,865 unmatched patients undergoing LAACCA or CA The data is presented as total (N) and percentages (%) for all categorical variables. Continuous data such as length of stay (LOS) and total hospital costs (THC) are summarized as mean with and without standard deviation (SD) †Adjusted for sex, elective vs. nonelective admission, age, race, primary payer, hospital region, obesity, dyslipidemia, coronary artery disease, obstructive sleep apnea, old PCI, old CABG, old MI, patient location, weekend admission, malignant neoplasm, dementia, chronic obstructive pulmonary disease, illness severity, concurrent use of anticoagulants, and Charlson comorbidity index. P-values are significant at values <0.05 * Adjusted mean difference aOR, adjusted odds ratio; CI, confidence interval; LOS, length of hospital stay; THC, total hospital costs; US$, United States dollars; LAACCA, combined left atrial appendage closure and catheter ablation; CA, catheter ablation alone; MI, myocardial infarction; AV, atrioventricular; SD, standard deviation; ICD, implantable cardioverter-defibrillators; CRT, cardiac resynchronization therapy; CABG, coronary artery bypass grafting; PCI, percutaneous coronary intervention

Outcomes	LAACCA (N = 27,945)	CA (N = 205,920)	aOR (95% CI)^ †^	P-value
Primary outcomes				
Inpatient mortality, n (%)	770 (2.8)	2,755 (1.3)	2.06 (1.67-2.55)	<0.001
LOS, days, mean	10	6	4.8*(4.5-5.0)	<0.001
THC, US$, mean (SD)	282,261 (5,085)	176,200 (2,081)	104,517* (95,990-113,044)	<0.001
Secondary outcomes, n (%)				
Cardiac tamponade	345 (1.2)	2,625 (1.27)	0.64 (0.49-0.84)	0.001
Acute MI	695 (2.5)	4,220 (2.0)	1.05 (0.85-1.30)	0.665
Complete AV block	1,880 (6.7)	11,040 (5.4)	1.38 (1.21-1.59)	<0.001
Sick sinus syndrome	1,940 (6.9)	13,855 (6.7)	1.08 (0.95-1.23)	0.228
Implantable device therapy (pacemakers, ICDs, CRT)	2,045 (7.3)	19,975 (9.7)	0.87 (0.76-0.99)	0.042
Pneumothorax	475 (1.7)	670 (0.3)	4.32 (3.13-5.99)	<0.001
Hemothorax	315 (1.1)	485 (0.2)	3.57 (2.34-5.43)	<0.001
Pneumonia	1,395 (5.0)	7,829 (3.8)	1.72 (1.47-2.02)	<0.001
Acute ischemic stroke	240 (0.9)	1,100 (0.5)	1.22 (0.87-1.73)	0.253
Phrenic nerve palsy	10 (0.03)	100 (0.05)	0.59 (0.12-2.87)	0.509
Hematoma, seroma, and hemorrhage	500 (1.8)	2,199 (1.1)	1.35 (1.05-1.76)	0.021
Thromboembolism	160 (0.6)	1,110 (0.5)	1.29 (0.86-1.91)	0.215
Femoral artery pseudoaneurysm	45 (0.2)	980 (0.5)	0.25 (0.12-0.52)	<0.001
Oxygen supplementation	655 (2.3)	8,180 (4.0)	0.74 (0.61-0.91)	0.005
Sepsis	480 (1.7)	2,200 (1.1)	1.89 (1.48-2.43)	<0.001
Acute renal failure	7,489 (26.8)	39,331 (19.1)	2.04 (1.89-2.21)	<0.001

In the overall propensity score-matched cohort, the mean duration of hospital stay was 8.2 days (SD 4.6), with 769 mortalities (2.1%) and a mean hospital charge of $236,946 (SD: $4,428). Approximately 509 (2.8%) mortalities occurred in the LAACCA cohort vs. 260 (1.4%) in the CA cohort (P < 0.001). Similar to the findings observed in the unmatched cohort of 233,865 hospitalizations (Table 2), hospitalizations in the LAACCA cohort were associated with significantly longer hospital stays (10.6 vs 5.6 days; P < 0.001), higher mortality (509 (2.8%) vs. 260 (1.4%); P < 0.001), and higher mean hospital costs (289,960 vs. 183,932; P < 0.001) in the propensity score-matched cohort (Table 3).

**Table 3:** Logistic regression model for clinical outcomes in 18,195 matched Patients undergoing LAACCA or CA The data is presented as frequency (N) and percentages (%) for categorical variables whereas continuous variables are presented as means with or without standard deviations * Significant at values <0.05 LOS, length of hospital stay; THC, total hospital costs; US$, United States dollars; CLCA, combined left atrial appendage occlusion and catheter ablation; CA, catheter ablation alone; MI, myocardial infarction; AV, atrioventricular; LAACCA, combined left atrial appendage closure and catheter ablation; CA, catheter ablation alone; ICDs, implantable cardioverter-defibrillators; CRT, cardiac resynchronization therapy

Outcomes	N (%)	N (%)	P-value*
	LAACCA (N = 18,195)	CA (N = 18,195)	
Primary outcomes			
Inpatient mortality, n (%)	509 (2.8)	260 (1.4)	<0.001
LOS, mean days	10.6	5.6	<0.001
THC, US$, mean (SD)	289,960 (4,375)	183,932 (2,772)	<0.001
Secondary outcomes			
Cardiac tamponade	230 (1.3)	385 (2.1)	0.010
Acute MI	520 (2.9)	455 (2.5)	0.013
Complete AV block	1,205 (6.6)	895 (4.9)	0.004
Sick sinus syndrome	1,250 (6.9)	1,170 (6.4)	0.155
Implantable device therapy (pacemakers, ICDs, CRT)	1,345 (7.4)	1,515 (8.3)	0.017
Pneumothorax	325 (1.8)	85 (0.5)	<0.001
Hemothorax	200 (1.1)	30 (0.7)	<0.001
Pneumonia	990 (5.4)	545 (3.0)	<0.001
Acute ischemic stroke	140 (0.8)	95 (0.5)	0.004
Phrenic nerve palsy	5 (0.0003)	5 (0.0003)	0.453
Hematoma, seroma, and hemorrhage	340 (1.9)	255 (1.4)	0.046
Thromboembolism	85 (0.4)	75 (0.5)	0.476
Femoral artery pseudoaneurysm	40 (0.2)	95 (0.5)	<0.001
Oxygen supplementation	475 (2.6)	590 (3.2)	0.341
Sepsis	315 (1.7)	180 (1.0)	<0.001
Acute renal failure	2,056 (11.3)	1,183 (6.5)	<0.001

Effect of LAACCA vs. CA on secondary outcomes in the propensity score-matched cohort

In the propensity score-matched analysis, outcomes were compared between the two matched cohorts: 18,195 patients who underwent LAACCA and 18,195 patients who underwent CA. Similar to results from the unmatched cohort, significant differences between LAACCA and CA cohorts were observed in terms of the development of cardiac tamponade (237 (1.3%) vs. 382 (2.1%); P = 0.010), acute myocardial ischemia (528 (2.9%) vs. 455 (2.5%); P = 0.013), complete atrioventricular block (1,201 (6.6%) vs. 892 (4.9%); P = 0.004), pneumothorax (328 (1.8%) vs. 91 (0.5%); P < 0.001), hemothorax (200 (1.1%) vs. 127 (0.7%); P < 0.001), pneumonia (983 (5.4%) vs. 546 (3.0%); P < 0.001), vascular access complications such as hematomas, seromas or hemorrhage (345 (1.9%) vs. 255 (1.4%); P = 0.046), Femoral artery pseudoaneurysm (36 (0.2%) vs. 91 (0.5%); P < 0.001), sepsis (309 (1.7%) vs. 182 (1.0%); P < 0.001), and the need for implantable device therapy (1,346 (7.4%) vs. 1,510 (8.3%); P = 0.017). No significant difference was observed in terms of the development of thromboembolism (73 (0.4%) vs. 91 (0.5%); P = 0.476), sick sinus syndrome (1,255 (6.9%) vs 1,164 (6.4%); P = 0.155), and need for oxygen supplementation (473 (2.6%) vs 582 (3.2%); P = 0.341). In addition, LAACCA was associated with a greater incidence of acute ischemic stroke (146 (0.8%) vs. 91 (0.5%); P = 0.004; Table 3).

## Discussion

To control more comprehensively for biases associated with the selection of a particular approach for AFib in patients with HF, we stringently matched LAACCA and CA patients using a propensity score-matching method. After matching, this study demonstrates that LAACCA is associated with poorer in-hospital outcomes, including higher inpatient mortality, longer duration of hospitalization, and higher hospital costs compared with CA alone. LAACCA was also associated with a higher incidence of post-procedure complications, including acute myocardial infarction, complete atrioventricular block, sick sinus syndrome, pneumothorax or hemothorax, pneumonia, acute ischemic strokes, and sepsis. On the other hand, CA was associated with a greater incidence of cardiac tamponade, femoral artery pseudoaneurysms, and subsequent need for implantable device therapy.

The growing clinical interest in the comparative effectiveness of LAACCA versus CA alone in patients with HF reflects recent advancements in the prevention and management of AFib, and highlights the importance of a focused analysis of treatment efficacy, patient safety, and healthcare outcomes [30,31]. LAACCA is being explored because of the limitations of CA alone in AFib management, especially in patients with HFrEF. Catheter ablation is a standard treatment for AFib and has been shown to improve symptoms and quality of life. CA has also been shown to improve ejection fraction and unplanned hospitalizations as well as reduce recurrences as compared with rate and/or rhythm control therapy [32,33]. However, its efficacy in patients with HF can be more complex because of the increased risk of thromboembolic events [34]. Left atrial appendage closure, typically used to reduce ischemic stroke risk in patients with AFib, is theorized to offer additional benefits in this patient group, particularly in managing the elevated risks associated with HF.

Das S et al. [35] present a similar investigation into the safety profile of LAAC in individuals with and without HF. The findings reveal a lack of substantial disparity in inpatient mortality and cardiac complications across both cohorts. However, similar to the findings of the index study, incidents of noncardiac complications, such as acute kidney injury and respiratory failure, were observed at an elevated rate within the HF group. While this investigation reinforces the safety of LAAC in patients with HF, it underscores the necessity of extended follow-up periods to develop more conclusive assessments.

Beyond the CHADS2-VASc risk profile of Afib patients, several factors can increase the risk of thromboembolism after CA. These include persistent or long-standing atrial fibrillation, which promotes left atrial thrombus formation; atrial enlargement and fibrosis, which disrupt normal atrial contraction; multiple prior ablations, which cause further atrial remodeling; and chronic kidney disease, which may affect anticoagulation efficacy. In these patients, LAAC may be beneficial, especially when long-term anticoagulation is contraindicated or not preferred, as it reduces stroke risk. Transcatheter LAAC is appropriate for patients with nonvalvular AF with high thromboembolic risk who are not suited for long-term oral anticoagulation (OAC) and who have adequate life expectancy (minimum >1 year) and quality of life to benefit from LAAC [36]. LAAC has been found to be comparable to oral anticoagulants after ablation in a recent study [37]. 

One of the earliest studies that compared LAACCA with CA alone in patients with AFib dates back to 2020 [38]. The results indicated that LAACCA was both safe and effective compared with either procedure performed in isolation. The combined procedure aimed to control heart rhythm and reduce the risk of ischemic stroke in patients with AFib. One of the key findings was that combined therapy in selected AFib patients, especially those with a prior history of stroke, was not only safe but also efficient. This efficiency was demonstrated in terms of reduced risks of ischemic stroke and bleeding following the procedure. Although the study findings are particularly significant because AFib is an independent risk factor for stroke, strokes associated with AFib tend to be more severe or fatal compared with non-AFib strokes [39], the findings of the analysis of matched cohorts in the index study have not reproduced these prior results.

The incidence of acute in-hospital strokes was found to be higher in patients receiving the combined procedure, whereas the incidence of thromboembolism was comparable between both groups. However, while catheter ablation for AFib has been effective in rhythm control and improving quality of life, no randomized clinical trial has shown a reduction in long-term ischemic stroke from CA alone. In contrast, LAAC with devices like the WATCHMAN™ has been shown in randomized trials to reduce strokes and can be an alternative to warfarin therapy for long-term ischemic stroke prevention [40-43]. The Abbott AMPLATZER™ Amulet™ (Abbott Laboratories, Chicago, USA) is an FDA-approved left atrial appendage closure device designed for stroke prevention in patients with Afib who are at high risk for thromboembolic events but cannot tolerate long-term anticoagulation therapy. It features a self-expanding, dual-layer design that offers enhanced stability and sealing ability. The device is delivered percutaneously, providing a minimally invasive option to reduce the risk of stroke by occluding the left atrial appendage [44].

The increased incidence of acute strokes in the index study may be attributable to a new thrombus forming on the left atrial face of the device and then potentially embolizing, or to a preexisting thrombus dislodged during LAACCA. While device-related thrombus is observed in some cases following LAAC, the majority (approximately 75%) of these thrombi do not result in stroke. Furthermore, only around 10% of strokes after LAAC can be directly attributed to device-related thrombus [45]. Therefore, it is essential to consider other potential causes of stroke following LAAC and avoid automatically attributing all strokes to device-related thrombus as a standard or default outcome. This approach ensures a more comprehensive understanding of stroke etiology in this patient population. 

In clinical practice, the detection of a thrombus within the left atrial appendage is often considered a contraindication to performing percutaneous left atrial appendage occlusion procedures [46]. Nevertheless, exceptional situations have been reported in the literature [47,48], where the necessity for left atrial appendage occlusion overrides the concerns associated with an existing thrombus. In these instances, the process of occluding the left atrial appendage carries an elevated risk of dislodging the thrombus. CA is widely recognized for its effectiveness in maintaining sinus rhythm and reducing AFib burden, potentially leading to a decreased risk of ischemic stroke. This could make its outcomes similar to those achieved with combined LAACCA [49]. However, despite extensive utilization and research data generation around CA, it has not been found to reduce the incidence of ischemic stroke.

Safety is a crucial consideration when combining these therapies. Each procedure carries its own set of risks, and understanding the cumulative risk profile is essential for patient safety and informed decision-making. In addition, the economic aspect of LAACCA versus CA alone is an important consideration. HF and AFib are associated with significant healthcare costs, and a combined approach has been associated with long-term cost-effective advantages, including up to 122 fewer disabling strokes, 203 fewer intracranial hemorrhages per 10,000 patients, and an incremental cost-effectiveness ratio of $11,072 per quality-adjusted life years compared with CA alone [50,51]. Although most research indicates that LAACCA is a cost-effective approach for ischemic stroke prevention in patients with AFib, questions about the reliability and validity of these findings persist because of concerns regarding the methodological quality of these studies. Furthermore, the literature indicates a rising trend in the use of LAACCA compared to CA alone, with both approaches showing similar 30-day readmission rates [52]. However, in the acute in-hospital setting, patients undergoing LAACCA can expect higher mean hospital costs and longer hospital stays compared with similar patients receiving CA alone.

Limitations

This comprehensive propensity score-matched analysis, focusing on in-hospital outcomes of LAACCA and CA among patients with HF, offers many useful insights. However, it is important to acknowledge certain limitations. First, the retrospective nature of the study means that although many cases were consecutive, this might not have been consistent across all participating hospitals. Second, even though a meticulous propensity score-matching method was used to adjust for initial differences between LAACCA and CA patient groups, the possibility of unaccounted confounding factors remains, potentially leading to residual treatment selection bias. Third, LAACCA can be performed in various sequences, either starting with CA or LAAC. Additionally, the choice of anesthesia, whether local or general, along with different puncture sites in the atrial septum can influence the approach and outcomes of the procedure.

These procedural variations in addition to residual patient-specific heterogeneity may have a significant impact on the success rates and complications of LAAC and CA. The NIS does not include provider notes, impressions, or postoperative documentation necessary for a definitive assessment of procedural success. However, procedural outcomes can be inferred indirectly through the incidence of complications and related interventions, such as the use of implantable devices for established complications, as demonstrated in the current study. Temporal relationships between observed complications could not be established due to reliance on retrospective data recorded using ICD-10 codes. This study analyses hospital data and does not explore long-term post-discharge outcomes. Future research should consider these factors when evaluating the comparative outcomes of combined LAACCA and CA alone.

In today’s environment of value-focused healthcare, it is crucial to thoroughly evaluate the clinical and economic implications of choosing LAACCA for each patient. This research contributes to the evolving evidence base, challenging previous claims of LAACCA’s superior postprocedural outcomes in patients with AFib and HF compared with a well-matched group undergoing only CA.

## Conclusions

This study is among the few that compare in-hospital outcomes of left atrial appendage closure combined with catheter ablation versus catheter ablation alone for treating atrial fibrillation in patients with heart failure. To the best of our knowledge, it is the only study that employs a propensity score-matching methodology within this cohort. The results indicate that combining left atrial appendage closure with catheter ablation does not lead to better in-hospital outcomes, including mortality, resource utilization, and acute post-procedure complications, compared to catheter ablation alone in managing atrial fibrillation among heart failure patients.
